# On the distribution and habitat use of the sub‐Antarctic fly *Hyadesimyia clausa* Bigot (Diptera, Tachinidae) according to citizen science

**DOI:** 10.1002/ece3.11169

**Published:** 2024-03-24

**Authors:** Rodrigo M. Barahona‐Segovia, Pablo R. Mulieri, Christian R. González, Felipe Osorio Zúñiga, Laura Pañinao‐Monsálvez

**Affiliations:** ^1^ Departamento de Ciencias Biológicas y Biodiversidad Universidad de Los Lagos Osorno Chile; ^2^ Moscas Florícolas de Chile Citizen Science Program Osorno Chile; ^3^ Consejo Nacional de Investigaciones Científicas y Técnicas (CONICET) Buenos Aires Argentina; ^4^ Museo Argentino de Ciencias Naturales ‘Bernardino Rivadavia’ (MACN) Buenos Aires Argentina; ^5^ Instituto de Entomología Universidad Metropolitana de Ciencias de la Educación Santiago Chile; ^6^ Escuela de Graduados, Instituto de Conservación, Biodiversidad & Territorio, Facultad de Ciencias Forestales & Recursos Naturales Universidad Austral de Chile Valdivia Chile

**Keywords:** biological interaction, habitat use, Magellanic peatland, mosses, Tachinidae, Wallacean shortfall

## Abstract

*Hyadesimyia clausa* Bigot is a morphologically striking tachinid that inhabits the Sub‐Antarctic Ecoregion of the Magallanes Region in Chile and Tierra del Fuego province in Argentina. Much of the distributional information about this species is restricted to the Cape Horn islands, which have extreme environmental conditions, but the species' natural history, range limits, and habitat use have never been described or confirmed. Our goals were to describe the distributional limits of this sub‐Antarctic fly with the help of citizen science and use this information type to describe this tachinid's habitat use and potential biological interactions with nonvascular and vascular flora. We found that citizen science significantly increased our understanding of the extent of occurrence, expanding the known distributional range by 195 km to the north and 153 km to the west. On the contrary, the values for the area of occupancy were not significant, but the occupancy overlap between different records was very low. We confirmed that *H. clausa's* habitat uses peatlands and although we have not provided evidence of pollination or movement of spores, we hypothesized, that the walking activity of *H. clausa* could help move sperm from mosses and pollen from the flowers of vascular plants, so they could act as potential pollinators. Citizen science can reduce and eliminate some scientific knowledge shortfalls and propose new ecological questions that could increase our knowledge of extreme ecosystems.

## Graphical abstract

Citizen science can fill the gaps in scientific knowledge for rare invertebrate species such as Hyadesimyia clausa. According to citizen science records, distribution increased from locality type to north and west of the Magallanes Region, we confirmed the use of peatlands and suggested potential biological interaction with vascular plants and mosses. Citizen science can address new ecological and biological questions, always identify their limits.

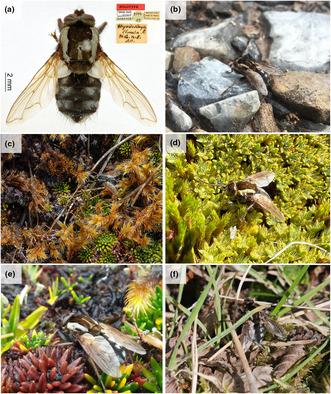

## 1 INTRODUCTION

**FIGURE 1:**
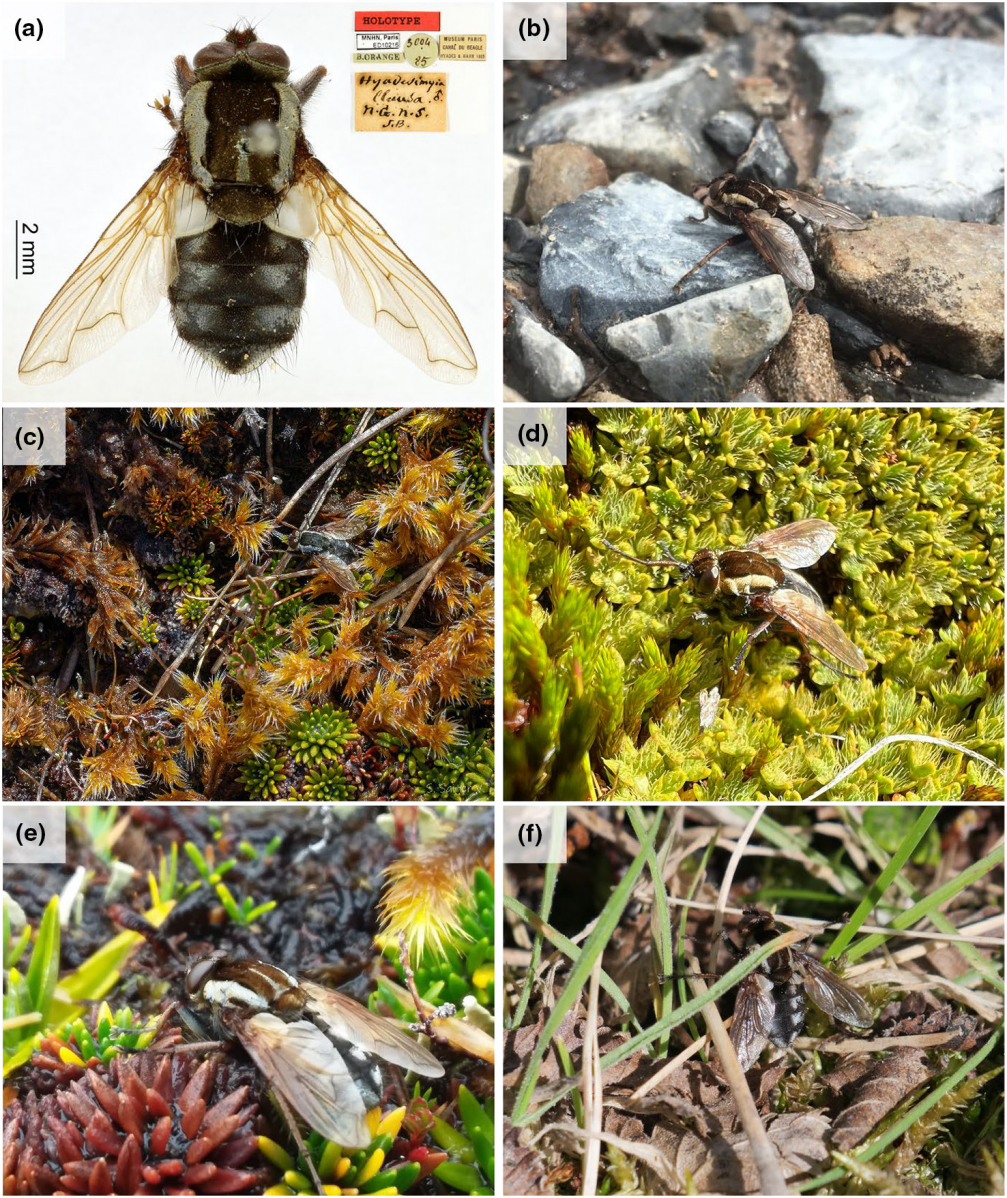
*Hyadesimyia clausa* Bigot, 1888: (a) holotype and labels courtesy of the MNHN (photo by Marion Depraetere, proyect RECOLNAT (ANR‐11‐INBS‐0004)); (b) the northernmost individual record, Torres del Paine National Park (photo by Francisco Ojeda); (c) individual walking through *Racomitrium* sp., *Donatia fascicularis* and *Myrteola nummularia*, Carlos III Island (photo by Sebastián Saiter); (d) individual walking through *Polytrichum strictum* and *Drapetes muscosa*, Ushuaia (photo by Michelle Locke); (e) individual walking through *Astelia pumilia*, *D. fascicularis* and *Racomitrium* sp., Riesco Island (photo by Raphael Forns); and (f) walking through *Racomitrium* sp. (photo by Gustavo Durán).

*Hyadesimyia clausa* Bigot (Diptera: Tachinidae) was described by Bigot (1888) in the *Mission scientifique du Cap Horn*, 1882*–*1883 based on a single specimen collected in Orange Bay, Hoste Island in the Tierra del Fuego Archipelago (O'Hara et al., 2021). Bigot named the species after Paul Daniel Jules Hyades, a naval physician who collected the first specimen while traveling in the Tierra del Fuego Archipelago (O'Hara et al., 2021). *Hyadesimyia clausa* belong to the Dexiinae subfamily and is a striking fly because of their distinctive coloration pattern and an unusual lack of acrostichal, dorsocentral, and intra‐alar setae (Figure 1a–f). The scutum coloration pattern is easily recognizable by its dark brown color with broad light‐gray margins and a median narrow strip of the same color from suture to scutellum. In addition, eight shiny gray and pollinose spots are distributed in the abdominal segments.

Brauer and Bergenstam (Brauer, 1889) considered the species to be part of the Oestridae family, probably based on its unusual lack of dorsal chaetotaxy. The same classification is followed by Brèthes (1907), Townsend (1936), and even Cortés (1973). Aldrich (1934) included a redescription and the first record of *H. clausa* from Argentina. Cortés (1986) included this species in a list of tachinids from the Magallanes Region (Chile). The distribution data for the species is scarce but seems to be associated with the far southern islands of the sub‐Antarctic Ecoregion (= Magellanic Subpolar Forest) and apparently associated with extreme ecosystems, according to the disparate records published (see Cortés, 1973). In addition, information regarding the hosts and habitat use of this species is completely absent from the literature. In this nature note, we provide new insights into the distribution and natural history of *H. clausa*, according to citizen science records. Our main goals were to (1) generate an updated description of its distribution, including the northernmost record for this species and (2) record the habitat use and discuss potential ecosystem services between *H. clausa* and other biotic components in peatlands and the flora of the sub‐Antarctic Ecoregion.

## 2 METHODS

### 2.1 Study site: Sub‐Antarctic Ecoregion

The sub‐Antarctic Ecoregion is one of the wildest places on the planet (Mittermeier et al., 2003). The retreat of the ice since the last glacial maximum has generated a large network of islands with navigable channels and well‐preserved biomes, such as beech forests, grasslands, and peatlands, which are still highly biodiverse, mainly with liverworts, mosses, and aquatic invertebrates, as well as unique environmental characteristics (Mansilla et al., 2012; Rosenfeld et al., 2023; Rozzi et al., 2008, 2012). Studies into the terrestrial invertebrate assemblages of these sub‐Antarctic biomes have mainly focused on extreme ecosystems, such as peatlands and subantarctic grasslands (see examples in Contador et al., 2020; Lanfranco, 1980, 1981, 1983) or *Nothofagus* forest (Lanfranco, 1977). The weather conditions include ambient temperatures between 3.1 and 6.9°C, mean rainfall of 460–1200 mm, and high UVA + UVB conditions (Schlatter & Riveros, 1997; Trest et al., 2015). In addition, some particular biological relationships in the sub‐Antarctic Ecoregion, such as the use by subantarctic mosses of coprophilous flies for reproductive purposes (Jofré et al., 2011), pose new ecological questions based on gaps in our knowledge of these biological processes, which have been poorly explored.

### 2.2 Citizen science dataset

To generate the most complete distributional overview of *H. clausa*, individual records were compiled from the collection of the Museo Argentino de Ciencias Naturales (MACN), the Canadian National Collection of Insects, Arachnids, and Nematodes (CNC); The Museu de Zoologia da Universidade de São Paulo, Brazil (MZUSP); The Muséum National d'Histoire Naturelle (MNHN) of Paris; The Natural History Museum of London (NHMUK); literature (Cortés, 1973, 1986), and *Moscas Florícolas de Chile* citizen science program (CS) from Facebook (https://www.facebook.com/groups/774986852548819) and iNaturalist (https://www.inaturalist.org/projects/moscas‐floricolas‐de‐chile). To find and match our target species with potential records, both in Chile and Argentina, we used the keywords “*Hyadesimyia*” (generic name) AND “Oestroidea” (superfamily name) OR “Tachinidae” (family name) in the search engine of Facebook and iNaturalist. In both cases, we filtered per year (between 2015 and 2023) to increase the probability of finding forgotten records or misidentified individuals and avoid temporal biases. Meanwhile, for iNaturalist, we filtered by the Aysén and Magallanes Regions for Chile and Tierra del Fuego Province for Argentina to optimize the search and find unclassified oestroid fly records according to historical distribution (Cortés, 1973). To standardize our search of records with CS, the corresponding author spent 1 hour every weekday for the 4 weeks of an entire month on each social media platform (i.e., Facebook and iNaturalist). Finally, from these citizen science datasets, we obtained for each photo (1) the most precise location, ideally with coordinates; (2) the date of the photo, (3) the photographic legacy, and (4) the original photo. In addition, the country and administrative region of the record, habitat characteristics, and a unique link for each photo (when possible) were added to the dataset (see Table S1).

To verify the taxonomic identification of our photographic records, morphological comparisons were conducted in three ways: (1) with morphological details such as wing venation, scutum chaetotaxy, and the coloration of mesonotum and metanotum that were provided by Aldrich (1934) and the complementary traits by Cortés (1973); (2) morphological characters comparison with photos of *H. clausa* housed in the MNHN (holotype) and NHMUK; and (3) finally, each photographic record was verified by a specialist (PRM). The morphological traits used are summarized in Table 1.

**TABLE 1:** Morphological traits used for the identification of photographic records of *Hyadesimyia clausa*.

Body part	Character	State
Scutum	Achrostichal setae	Absent
	Dorsocentral setae	Absent
	Intra‐alar setae	Absent
Mesonotum and scutellum	Coloration pattern	Dark Brown, with broad light‐gray margins and a median narrow stripe (from suture to scutellum)
Wing	Vein M	M ending in R_4 + 5_ before wing margin (short petiole)
	Coloration pattern	Inferior margin of the wing hyaline
Abdomen	Spot coloration and pattern	Shiny gray pollinose spots in the lateral margins of the first four tergites

### 2.3 Data analysis and visualization

To demonstrate the contribution of citizen science toward enlarging the known area of distribution of *H. clausa*, we calculated the extent of occurrence (EOO) as a classic convex minimum polygon, which was defined as the sum of all internal angles higher than 180° (IUCN, 2012). To test whether citizen science could expand the known area of distribution, we separated the EOO into two groups: (i) historical records from literature and museums, and (ii) then, all CS records. Both datasets were compared with a chi‐squared test for given probabilities. In parallel, we also calculated the areas of occupancy (AOO). It was defined as the sum of all the 2 × 2 km quadrants where our fly target species had been recorded and represented the realized niche known as *H. clausa*. The AOO datasets also were separated into (a) historical (i.e., literature + museum) and (b) the CS records. AOOs data were also compared using a chi‐squared test for given probabilities. The distribution metrics, such as EOOs and AOOs, were calculated using the *ConR* package in the R software (Dauby et al., 2017). A distribution map with the peatland layer was created with ArcGIS (ArcMap) v.10.8 and the cadaster of native vegetation of CONAF‐Conama‐BIRF (2015). All statistical analyses were performed in R software v.2022.07.02 (R Development Core Team, 2023).

## 3 RESULTS

### 3.1 Distribution

**FIGURE 2:**
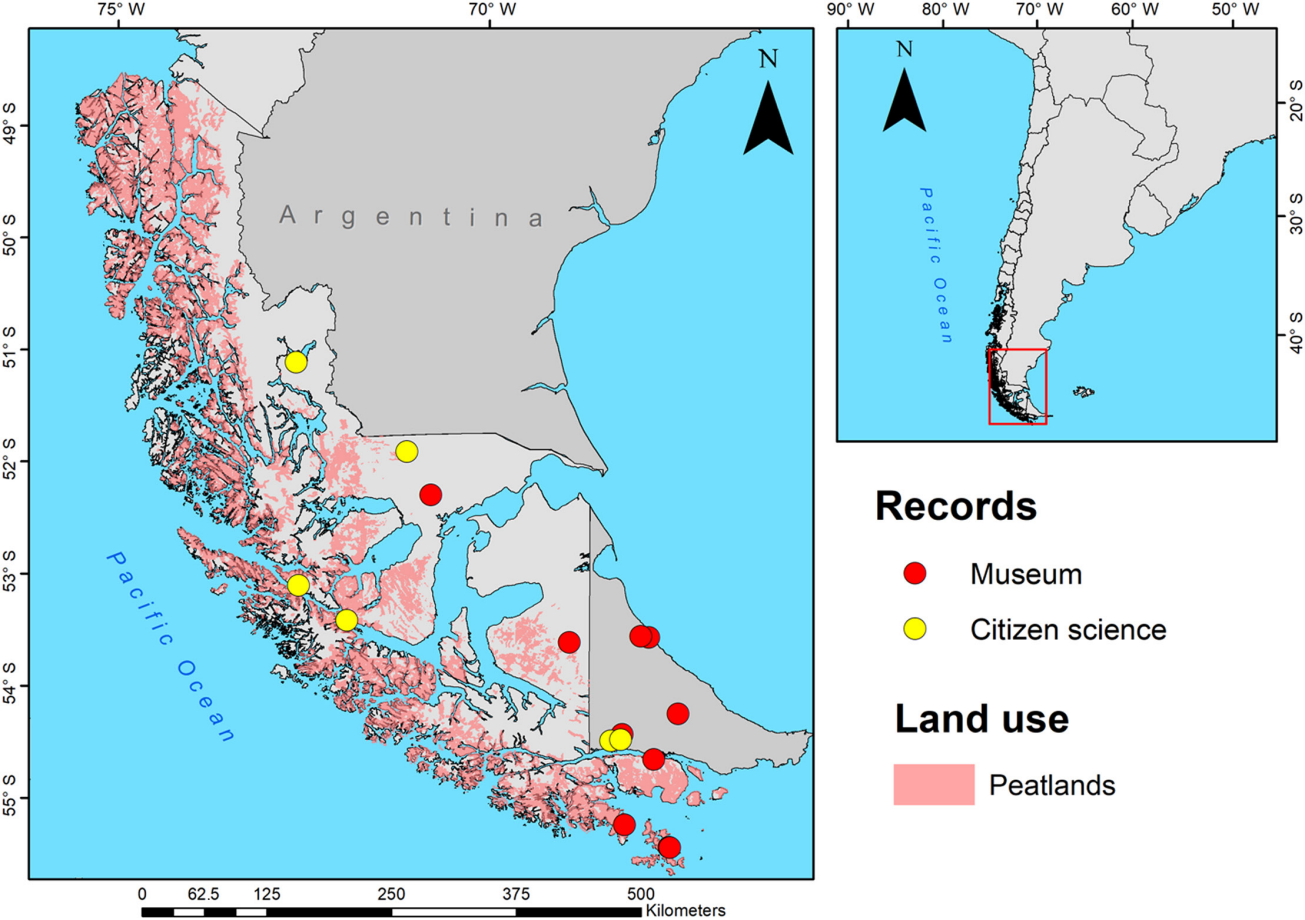
Distribution map of *Hyadesimyia clausa* Bigot, 1888 in the sub‐Antarctic Ecoregion (Magellanic Subpolar Forest). Peatland distribution (pink color) is only available for the Chilean territory.

We added six new records to the distribution records for *H. clausa* in Argentina and Chile (Table 2; Figure 2). The northern limit of the range of *H. clausa* increased by 195.50 km from Monte Alto to the Torres del Paine National Park and this is now the most boreal record known for the species (Figure 1b). The southwestern distribution also increased by 153 km from Monte Alto to the western coast of Riesco Island (Figure 2). The historical EOO and AOO were 21,276 and 36 km^2^ with nine subpopulations, whereas the EOO and AOO obtained through just CS were 46,113 and 28 km^2^ with seven subpopulations. We found statistical differences between the historical and citizen science EOOs (*χ* ^2^ = 9154, df = 1, *p* < .0001), but we did not find different areas of occupancy between the two subsets of records (*χ* ^2^ = 1, df = 1, *p* < .3173). However, there were a few overlaps between the occurrences from the CS and historical datasets (Figure 2). Combining the EOOs, the total distribution covered by *H. clausa* was 79,614 km^2^ (including the sea area) and the AOO was 52 km^2^.

**TABLE 2:** Records of *Hyadesimyia clausa* Bigot from Argentina (Tierra del Fuego) and Chile (Magallanes).

Locality	Province/region	Latitude	Longitude	Date	Legacy	Source
Orange Bay, Hoste Island	Magallanes	−55.5316	−68.0347	No date	Paul D.J. Hyades & Hahn	Bigot (1888), MNHN^a^
Río Grande	Tierra del Fuego	−53.8366	−67.7038	No date	P.W. Reynolds	Aldrich (1934), NHMUK
Tierra Mayor	Tierra del Fuego	−54.7162	−68.0864	January 25, 1960	Sixto Coscaron	MACN
Caleta Lientur	Magallanes	−55.7272	−67.3071	August 22, 1960	D. Lanfranco	MZUSP
Puerto Williams	Magallanes	−54.9361	−67.5887	November 22, 1960	L.E. Peña	CNC
Estancia Cameron	Magallanes	−53.8881	−68.9118	January 12, 1966	E. Schlinger & M. Irwin	Cortés (1973)
Monte Alto	Magallanes	−52.5452	−70.9633	December 17, 1976	D. Lanfranco	Cortés (1986)
Scourfield Bay	Magallanes	−55.7242	−67.3169	February 25, 1980	D. Lanfranco	Cortés (1986)
Caleta Toledo, Deceit Island	Magallanes	−55.5308	−68.0357	December 3, 1986	D. Lanfranco	Cortés (1986)
Fagnano Lake	Tierra del Fuego	−54.5224	−67.2253	01–1999	Juan Carlos Mariluis	MACN
Riesco Island	Magallanes	−53.3094	−72.9880	April 1, 2019	Raphael Forns	CS
Torres del Paine National Park	Magallanes	−51.3001	−72.8436	December 12, 2019	Francisco Ojeda	CS
Carlos III Island	Magallanes	−53.6454	−72.2845	October 29, 2021	Sebastián Saiter	CS
Ushuaia	Tierra del Fuego	−54.7695	−68.1887	October 13, 2022	Federico Muñoz	CS
Ushuaia	Tierra del Fuego	−54.7749	−68.3403	February 21, 2023	Michelle Locke	CS
Lapataia, Ushuaia	Tierra del Fuego	−54.8230	−68.5677	October 15, 2023	Gustavo Durán	CS

^a^ Holotype.

### 3.2 Habitat use and natural history

The phenology of this tachinid fly extended from October to March, with a peak in the austral summer months (December and January). Four records showed *H. clausa* adults that had been photographed while walking in Magellanic peatlands. Photos revealed that these peatlands were covered with *Racomitrium* sp., *Donatia fascicularis* J.R. Forst. & G. Forst. and *Myrteola nummularia* (Poir.) O. Berg (Figure 1b–d); *Polytrichum strictum* Bridel, J. Bot. (Schrader) and *Drapetes muscosa* Banks ex Lam. (Figure 1c), or *Astelia pumilia* (G. Forst.) Gaudich. (Figure 1d). Meanwhile, other records show *H. clausa* inhabiting grasslands and areas influenced by bodies of water such as lakes and flood‐prone areas (Figure 2).

## 4 DISCUSSION

Scarcely any examples of Tachinids—particularly *H. clausa*—were found during a long‐term sampling of the Magellanic Forest, transitional habitats, and peatlands conducted by Lanfranco (1977, 1980, 1981, 1983; except for the records in Table 1). However, the great richness of peatland species stands out, as well as their influence on neighboring communities (Lanfranco, 1981). According to Cortés (1986), a female *H. clausa* was collected on peat from Deceit Island. This clue was the only information about the natural history of *H. clausa*. Our study has made it possible to confirm the habitat use of peatlands by *H. clausa*.

Information on the natural history and ecology of flies is scarce; however, citizen science can decrease and help close the gaps generated by Wallacean shortfall (i.e., gaps in distribution records; Hortal et al., 2015). As in many other taxa, citizen science has contributed towards decreasing the shortfall in spatial occurrences for thousands of species, and flies are no exception. Our knowledge of the extent of occurrence of both tiny and inconspicuous flies as well as large and striking flies has increased thanks to biodiversity platforms such as iNaturalist and Facebooks groups devoted to flies (e.g., Barahona‐Segovia et al., 2023; Clem et al., 2023; Jaume‐Schinkel, Kvifte, et al., 2023, Jaume‐Schinkel, Mengual, et al., 2023). Furthermore, Clem et al. (2023) demonstrated that citizen science can be a key player in expanding the known range of flies at the local level. Here, the northern limit of *H. clausa—*an endemic fly of the Sub‐Antarctic Ecoregion—has extended from the Cape Horn Archipelago to the Torres del Paine National Park. Likely, this tachinid fly species could even be found in the peatlands in the Aysén Region of Chile and the Santa Cruz province of Argentina. Therefore, we encourage the use of citizen science when it is pertinent while advising the need for caution about the limitations of this method for some dark taxa (e.g., phorids, mycetophilids), as well as poor‐quality photos, technological, temporal, and geographic biases.

Climate change impacts peatlands such as decreasing the moss cover and accelerating the flowering of vascular plants (Antala et al., 2022). If the biology of *H. clausa* is closely related to peatlands—something we still need to explore further—the impacts of global warming, for example, can reduce the total area of mosses available for this fly species, increasing their extinction risk. Furthermore, the sooner flowering of shrub plants in peatlands could reduce the probability of *H. clausa* obtaining food (nectar) from potential interactions, and eventually, affect the same time potential ecosystem services of this tachinid in this particular plant community.

Jofré et al. (2011) determined that some moss species recruit coprophilous flies such as Muscidae and Tachinidae fly species to use as vectors, dispersing their spores. This function has also been described by Cronberg et al. (2006) and Cronberg (2012), who determined that some microarthropods like springtails and mites could move sperm from mosses, helping to fertilize them. Regarding this, although we have not provided evidence of pollination or movement of spores, we hypothesized that the walking activity of *H. clausa* on mosses could perform the sporophytes dispersion. In addition, tachinids are commonly observed feeding on flowers and acting as potential pollinators (Stireman III et al., 2006). Therefore, it is likely that *H. clausa* could also participate in the pollination of peatland vascular plant species observed by citizen scientists. Sporophytes presence and the flowering of some vascular plants observed in peatlands in the austral summer (December–February; Jofré et al., 2010), coincides with the most frequent season of *H. clausa*, reinforcing our hypothetical idea of pollination services. Further studies to test *H. clausa* as a vector and pollinator of the peatland community and raise the importance (or not) of this tachinid.

We concluded that citizen science records can fill in gaps in knowledge, such as geographic distribution, and at the least, suggest potential interactions with vascular plants and mosses. Further systematic studies are needed to address new questions in ecology, complement the current information known (as the case of coprophilous flies and *T. dubyi*), and increase the knowledge of the ecosystem services provided not only by *H. clausa* but also by all peatland communities of insects.

## AUTHOR CONTRIBUTIONS

**Rodrigo M. Barahona‐Segovia:** Conceptualization (equal); funding acquisition (equal); investigation (equal); methodology (equal); project administration (equal); resources (equal); visualization (equal); writing – original draft (equal); writing – review and editing (equal). **Pablo R. Mulieri:** Investigation (equal); validation (equal); writing – original draft (equal); writing – review and editing (equal). **Christian R. González:** Conceptualization (equal); investigation (equal); validation (equal); visualization (equal); writing – review and editing (equal). **Felipe Osorio Zúñiga:** Conceptualization (equal); investigation (equal); validation (equal); visualization (equal); writing – review and editing (equal). **Laura Pañinao‐Monsálvez:** Investigation (equal); methodology (equal); visualization (equal); writing – review and editing (equal).

## FUNDING INFORMATION

RMBS was supported by the Agencia Nacional de Investigación y Desarrollo (ANID), grant [SIA 85220045], and FOZ was supported by Fondo Nacional de Desarrollo Científico y Tecnológico (FONDECYT) grant PhD [21231650].

## CONFLICT OF INTEREST STATEMENT

The authors declare that they have no conflicts of interest that influence this study.

## Supporting information


Table S1

## Data Availability

The data that supports the findings of this study are available in the Supporting Information for this article.
