# Supplementary material for: On the distribution and habitat use of the sub‐Antarctic fly Hyadesimyia clausa Bigot (Diptera, Tachinidae) according to citizen science
Source: Ecol Evol. 2024 Mar 24;14(3):e11169. doi: 10.1002/ece3.11169 (PMC10961473; doi:10.1002/ece3.11169)
Supplement: Supplementary file 1 — Table S1 [file ECE3-14-e11169-s001.zip › legends.docx]

Supplementary Table S1: Records of *Hyadesimyia clausa* Bigot from Argentina (Tierra del Fuego) and Chile (Magallanes). Acronyms used: MACN = Museo Argentino de Ciencias Naturales; the Canadian National Collection of Insects, Arachnids, and Nematodes (CNC); The Museu de Zoologia da Universidade de São Paulo, Brazil (MZUSP); The Muséum National d'Histoire Naturelle (MNHN) of Paris; and The Natural History Museum of London, UK (NHMUK).
